# Omega-3-carboxylic acids provide efficacious anti-inflammatory activity in models of crystal-mediated inflammation

**DOI:** 10.1038/s41598-018-19252-x

**Published:** 2018-01-19

**Authors:** Cory Iverson, Andrew Bacong, Sha Liu, Scott Baumgartner, Torbjörn Lundström, Jan Oscarsson, Jeffrey N. Miner

**Affiliations:** 1 Ardea Biosciences, 9390 Towne Centre Drive, San Diego, California USA; 2 AstraZeneca Gothenburg, Global Medicines Development, SE-43183 Mölndal, Sweden

## Abstract

This study assesses the efficacy and exposure–response relationship of omega-3-carboxylic acids (OM-3 CA) in models of crystal-based inflammation. Human THP-1 macrophages and primary peripheral blood mononuclear cells exposed to multiple inflammatory crystal types were used to determine the anti-inflammatory potential of omega-3 (OM-3) fatty acids *in vitro*. Anti-inflammatory effects of OM-3 CA *in vivo* were tested in rat monosodium urate (MSU) crystal air pouch and rat knee intra-articular MSU injection models. Acute treatment with the OM-3 fatty acid docosahexaenoic acid suppressed MSU-, cholesterol crystal-, and calcium pyrophosphate crystal-mediated interleukin-1β (IL-1β) production *in vitro*. *In vivo*, OM-3 CA dose-dependently reduced crystal-mediated cell migration, exudate volume, and levels of IL-1β and prostaglandin E_2_. Following intra-articular injection of MSU, treatment with OM-3-CA (1 mL/kg) and indomethacin (1 mg/kg) resulted in similar mean reductions in pain (23% and 41%, respectively) and swelling (58% and 50%, respectively), compared with controls. Additionally, in complex formulations of OM-3 fatty acids, high levels of palmitic acid could reduce the *in vivo* effect on crystal-mediated IL-1β elevation. OM-3 CA has a broadly efficacious anti-inflammatory effect with a strong exposure–response relationship that could be beneficial in prevention and treatment of crystal arthritis, with potential applications in other IL-1β-mediated diseases.

## Introduction

Omega-3 (OM-3) fatty acids, mainly docosahexaenoic acid (DHA) and eicosapentaenoic acid (EPA), have two main and mechanistically distinct clinical activities: lipid lowering, especially of serum triglyceride-rich lipoproteins^1^, and modulation of inflammatory activity via multiple mechanisms^2–5^. OM-3 fatty acid formulations have been studied in inflammatory diseases such as rheumatoid arthritis (RA), inflammatory bowel disease, asthma, and cardiovascular disease^6–12^. The rationale for studies in RA was based on reduction in pro-inflammatory prostaglandin (PG) synthesis mediated by OM-3 fatty acids^13^. A meta-analysis of data for OM-3 fatty acid-based therapies in RA clinical trials showed modest but consistent improvements in joint swelling and pain, and reduced use of non-steroidal anti-inflammatory drugs (NSAIDs)^14,15^. Studies have also shown that OM-3 fatty acids can inhibit production of pro-inflammatory cytokines such as tumour necrosis factor α, interleukin (IL)-1β, IL-8, and IL-6, probably via reduced nuclear factor kappa B activity mediated by upstream modulation of Toll-like receptor 2/4 signalling^16,17^. More recently, two additional immune modulatory properties have been ascribed to DHA and EPA: formation of specialized pro-resolving mediators, such as the resolvin series of metabolites, and blockade of activation of the NLRP3 inflammasome, resulting in a reduction in IL−1β production^18,19^.

These anti-inflammatory activities are involved in the response to crystal deposition, suggesting that OM-3 fatty acid therapy could be useful for the prevention and treatment of arthritis caused by monosodium urate (MSU) and other crystals^20,21^. To the best of our knowledge, no randomized, controlled clinical studies have assessed the efficacy of OM-3 fatty acid-based therapies for the prevention or treatment of crystal-induced inflammation, such as acute gout. However, a case-control study showed that low omega-3 fatty acid levels were associated with more frequent gout attacks^22^. Additionally, few *in vivo* studies have demonstrated activity of OM-3 fatty acids in models of crystal-induced inflammation^23^, and none have shown an exposure–response relationship. Gout-induced inflammation is characterized by pain, swelling and heat, typically in the synovium of the great toe. The rat MSU air pouch model mimics the synovial space^24,25^, and allows the measurement of white blood cell (WBC) infiltration, exudate production, and the production of pro-inflammatory molecules in response to MSU crystals. The rat air pouch model is limited to an acute challenge because inflammation occurs during a 4–6 hour timeframe. We also used the rat intraarticular MSU injection model to follow the progression of inflammation by assessing pain and swelling caused by MSU for multiple days through to its self-resolution^26,27^. Pain was quantified in the rats by mechanical allodynia, another valuable endpoint of the utmost concern for clinical treatment of acute gout flares. Clinical studies have found that high purity, large quantity, and high bioavailability of particular OM-3 fatty acids were required to lower triglyceride levels effectively^28,29^. Therefore, we hypothesized that this would also be the case with OM-3 fatty acid-based therapies for inflammation. The notion of an ‘anti-inflammatory threshold’ of DHA or EPA exposure has been proposed and is consistent with clinical failures of low-purity, low-dose OM-3 fatty acid supplementation in RA studies^15^.

Epanova® (omega-3-carboxylic acids [OM-3 CA]; AstraZeneca) is a mixture of free carboxylic acids (enriched in EPA and DHA, and with low levels of saturated fatty acids) that has received US Food and Drug Administration approval for the treatment of severe hypertriglyceridaemia. OM-3-carboxylic acids are more bioavailable under fasting conditions than the corresponding ethyl esters because an activated pancreatic lipase is not required for intestinal absorption of the free acids^30^. In this study, we used rat models of acute gout flare to assess the efficacy and exposure–response relationship of OM-3 CA on crystal-mediated inflammation *in vivo*. Additionally, human THP-1 macrophages and primary peripheral blood mononuclear cells exposed to multiple inflammatory crystal types were used to determine the anti-inflammatory potential of omega-3 fatty acids *in vitro*.

## Methods

### Reagents

Purified eicosapentaenoic acid and docosahexaenoic acid, palmitic acid, and oleic acid were obtained from Sigma Aldrich. Epanova® (omega-3-carboxylic acids [OM-3 CA]) was supplied by AstraZeneca. Over-the-counter omega-3-triglyceride supplements (Nordic Naturals) were purchased from Amazon. Safflower oil was purchased from a commercial grocer. Colchicine, indomethacin, and Ac-YVAD-cmk (YVAD) were purchased from Sigma Chemical. MSU, cholesterol, and calcium pyrophosphate crystals were made according to established protocols^31,32^. Crystals were tested and found to be free of endotoxins using PYROGENT™–5000 Kinetic Turbidimetric Limulus Amebocyte Lysate assays (Lonza). Cell-culture supernatant and pouch exudates were analysed for IL-1β content with electrochemiluminescence enzyme-linked immunosorbent assay (ELISA) plates (Mesoscale). Prostaglandin E_2_ (PGE_2_) content was analysed using a competitive enzyme immunoassay (R&D Systems).

### Cell culture and primary cell isolation

*In vitro* experiments were performed using THP-1 (ATCC, Manassas, VA) cells differentiated to THP-1 macrophages and peripheral blood mononuclear cells (PBMCs) that were freshly isolated from healthy human donors. Cells were maintained in Roswell Park Memorial Institute medium with 10% fetal bovine serum (FBS) at 37 °C with 5% CO_2_, but this was switched to low-serum Opti-MEM™ medium (Invitrogen) for stimulation experiments. THP-1 monocytes were differentiated into macrophages for 3 hours with 200 nM phorbol 12-myristate 13-acetate and then left to recover overnight in 10% FBS containing media prior to crystal treatments. PBMCs were separated from red blood cells by a 10-minute room-temperature centrifugation at 500 × *g* using heparinized whole blood layered onto Histopaque 1077 (Sigma Aldrich). The PBMC band was recovered and washed twice with phosphate-buffered saline (PBS) before cells were counted, and used immediately for crystal stimulation studies.

### Crystal stimulation studies

THP-1 macrophages were plated in a 96-well plate (2 × 10^4^ cells per well). Cells were treated with drug or vehicle (0.5% ethanol) for 1 hour prior to a 24-hour incubation with drug or vehicle and crystal. For the fatty acid interaction studies, each selected fatty acid was incubated for a total of 26 hours with differentiated THP-1 cells. Primary cells from five individual donors were plated in a 96-well plate (1 × 10^5^ cells per well). Primary PBMCs were primed for 1 hour with lipopolysaccharide prior to treatment for 6 hours with drug and crystal. Cell supernatants were analysed for IL-1β using a chemiluminescence ELISA (Meso Scale Discovery), and PGE_2_ levels were analysed via ELISA (R&D Systems).

### Fatty acid quantification

Dosing solution and serum fatty acid concentrations were analysed using gas chromatography with flame ionization detection at OmegaQuant (Sioux Falls, SD, USA). Fatty acids were identified by comparison with a standard mixture of fatty acids (GLC OQ-A, NuCheck Prep, Elysian, MN, USA), which was also used to determine individual fatty acid calibration curves. The di-C17:0 PL was used to calculate the recovery efficiency of the assay and was applied to all fatty acids. Detailed methods can be found in Maki *et al*.^33^.

### Animal studies

All standard operating procedures involving animals were carried out in accordance with the ‘Institutional Animal Care and Use Committee Guidebook’ published by the Office of Laboratory Animal Welfare. All experimental protocols were approved by the Institutional Animal Care and Use Committee of Washington Biotechnology, Inc.

Study rats were maintained on Teklad Global 18% Protein Rodent Diet (Harlan Laboratories) and provided with water *ad libitum*. The rats were divided into 4 groups of 10 rats, receiving either safflower oil vehicle, OM-3 CA, or omega-3-triglycerides. Each group had a similar mean weight at the start of the study. MSU air pouch studies were performed using male Sprague Dawley rats weighing 160–180 g that had 30 mL of sterile air injected subcutaneously for 6 days (AIR ARD-20, AIR ARD-24 and AIR ARD-26)^34^. On the day of the experiment, 150 mg of MSU in 15 mL of PBS, or PBS only (as a control), was injected into the air pouch^35^. Fatty acid solutions tested had purities of >99% and a density of 0.9 g/mL, and were orally dosed by volume from 0.1 mL/kg up to 3.0 mL/kg. Solutions of safflower oil vehicle, OM-3 CA, or omega-3-triglycerides were dosed daily by oral gavage for 7 days prior to MSU injection; colchicine (0.5 mg/kg) was given by subcutaneous injection 2 hours prior to MSU pouch injection. Exudate and white blood cell counts were performed 4 hours after the MSU injection.

In the knee-injection model, 2 mg of MSU in 50 μl of PBS was injected intra-articularly into the left knee of male Wistar rats weighing 250–300 g (IA ARD-1, IA ARD-2), and the animals were studied for 96 hours (4 days). Mechanical allodynia thresholds and knee diameters were then measured daily for 4 days^27^. Animals were dosed daily by oral gavage for 7 days with a safflower oil vehicle, indomethacin (1.0 mg/kg), or OM-3 CA prior to MSU injection. The dosing of vehicle, OM-3 CA, and indomethacin continued through the 4-day observational post-MSU injection period.

### Statistics

Graphpad and Microsoft Excel software were used to generate graphs and perform statistical analyses. Student’s *t*-test was used for *in vitro* comparisons between treated and untreated samples. Kruskal–Wallis non-parametric tests were used to compare exudate IL-1β and PGE_2_ endpoints between groups, followed by Dunn’s multiple comparison tests when appropriate. One-way analyses of variance, with either Dunnett’s or Bonferroni’s multiple comparison tests when appropriate, were used to evaluate treatment effects across groups. Non-linear fit regression analyses were performed on OM-3 CA dose groups 0.03–1.0 mL/mg with no constraints, and variable slope was used to generate the exposure–response half-maximal inhibitory concentration (IC_50_) and the coefficient of determination (*R*^2^).

## Results

### OM-3 fatty acids preferentially modulate crystal-induced IL-1β production *in vitro*

Resident macrophage recognition of disease-associated crystals and subsequent release of pro-inflammatory mediators has been established as the primary inflammatory event leading to gout and pseudo-gout flares^36,37^. This process is mediated through Toll-like receptor 2/4-dependent cell priming, NLRP3 inflammasome activation, and release of mature IL-1β. MSU also upregulates cyclooxygenase-2 expression and induces subsequent PG release^38^.

**Figure 1:**
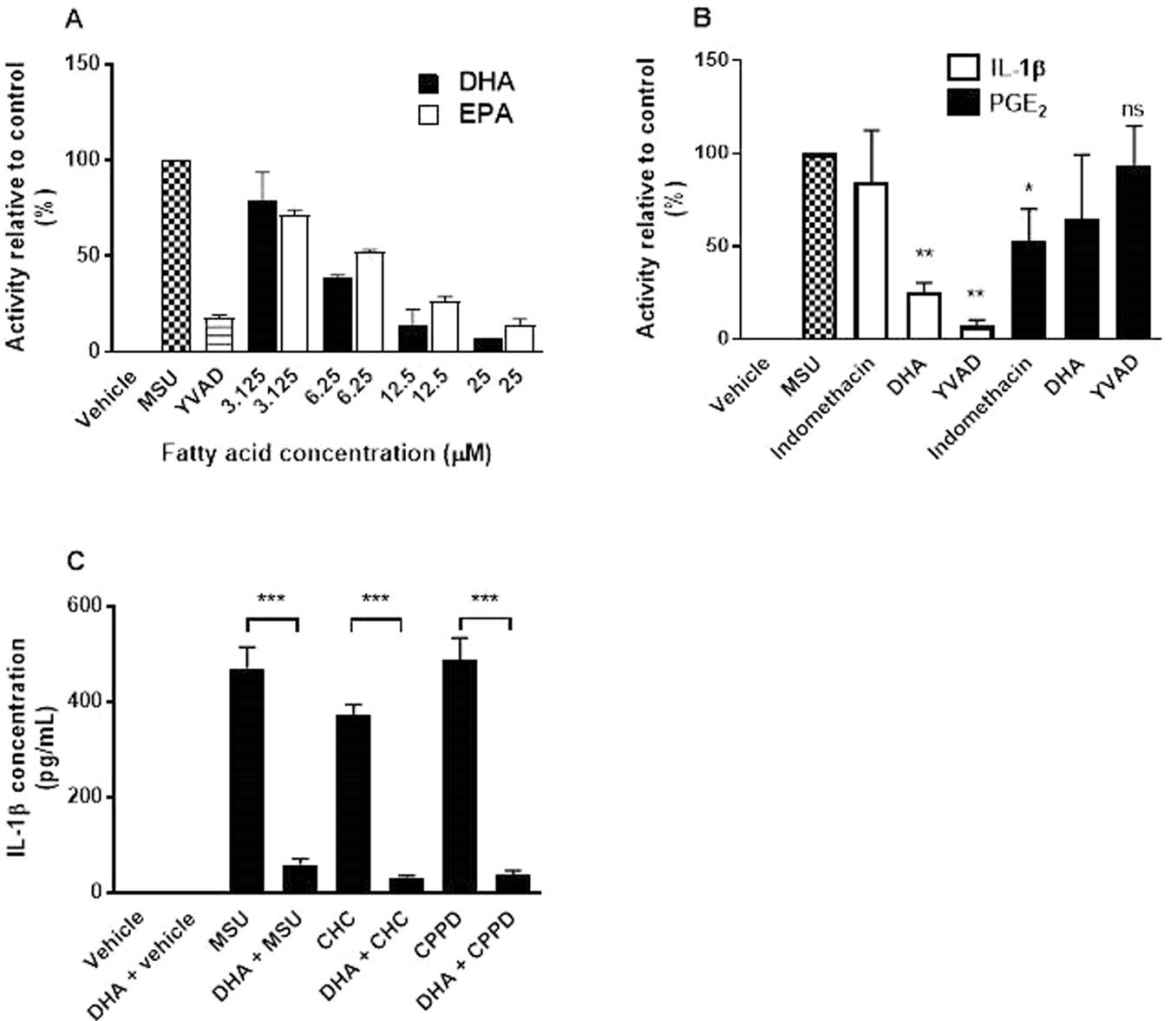
*In vitro* cell-based activity of long-chain omega-3 DHA and EPA in crystal-mediated inflammation. (**A**) Dose titration of DHA and EPA in THP-1 macrophages (n = 2) alongside YVAD, MSU and vehicle control treatments (n = 8). (**B**) MSU checked bar indicates maximal induction of either IL-1β or PGE_2_ from the same experiment; DHA (20 µM) and YVAD (10 µM) preferentially blocked MSU-induced IL-1β, and indomethacin preferentially blocked PGE_2_. (**C**) In human PBMCs, DHA (20 µM) was used to block IL-1β production induced by 0.2 mg/mL of MSU, 5 mg/mL of CHC and 5 mg/mL of CPPD crystals. Error bars indicate the standard error of the mean. THP-1 data from three independent experiments and PBMC data from five individual donors. *P < 0.05, **P < 0.01, ***P < 0.001 versus crystal using *t*-tests. ns, not significant versus MSU. CHC: cholesterol; CPPD: calcium pyrophosphate; DHA: docosahexaenoic acid; EPA: eicosapentaenoic acid; IL-1β: interleukin-1β; MSU: monosodium urate; PBMC: peripheral blood mononuclear cell; PGE_2_: prostaglandin E_2_.

DHA and EPA reduced production of IL-1β in MSU-stimulated THP-1 macrophages in a dose-dependent manner, with maximal inhibition similar to that produced by the peptide caspase 1 inhibitor YVAD. DHA and EPA potencies were similar, IC_50_ 4.6 µM and 6.0 µM, respectively (Fig. 1A). This finding confirms reports of OM-3 *in vitro* activity in a similar model, which used nigericin as an NLRP3 agonist^5^. To evaluate the effects on PG synthesis mediated by MSU, THP-1 macrophages were pre-incubated for 1 hour with DHA (20 μM), YVAD (10 μM), or indomethacin (20 μM), and then stimulated with MSU (0.2 mg/mL). After 24 hours, IL-1β and prostaglandin E_2_ (PGE_2_) levels in the media were measured. YVAD blocked production of IL-1β but not PGE_2_. The opposite effect was observed for the cyclooxygenase-1/2 inhibitor indomethacin. The use of these controls showed no feed-forward stimulation of either molecule by the other in this experiment. DHA preferentially suppressed production of IL-1β, having little effect on PGE_2_ induction (Fig. 1B).

Cholesterol and calcium pyrophosphate (CPP) crystals have been shown to activate NLRP3 signalling in a similar manner to MSU, albeit at different concentrations (MSU crystals are strongly immunostimulatory, requiring a concentration of only 0.2 mg/mL to achieve a similar level of IL-1β induction to 5 mg/mL of the other crystal types), and with differences in the overall intensity of the IL-1β response^31,32^. Purified DHA potently inhibited MSU, cholesterol and CPP crystal-mediated IL-1β production in human primary PBMCs primed with lipopolysaccharide (Fig. 1C).

### OM-3 CA reduces crystal-mediated cell migration, exudate production, IL-1β induction, and PGE_2_ induction *in vivo*

**Figure 2:**
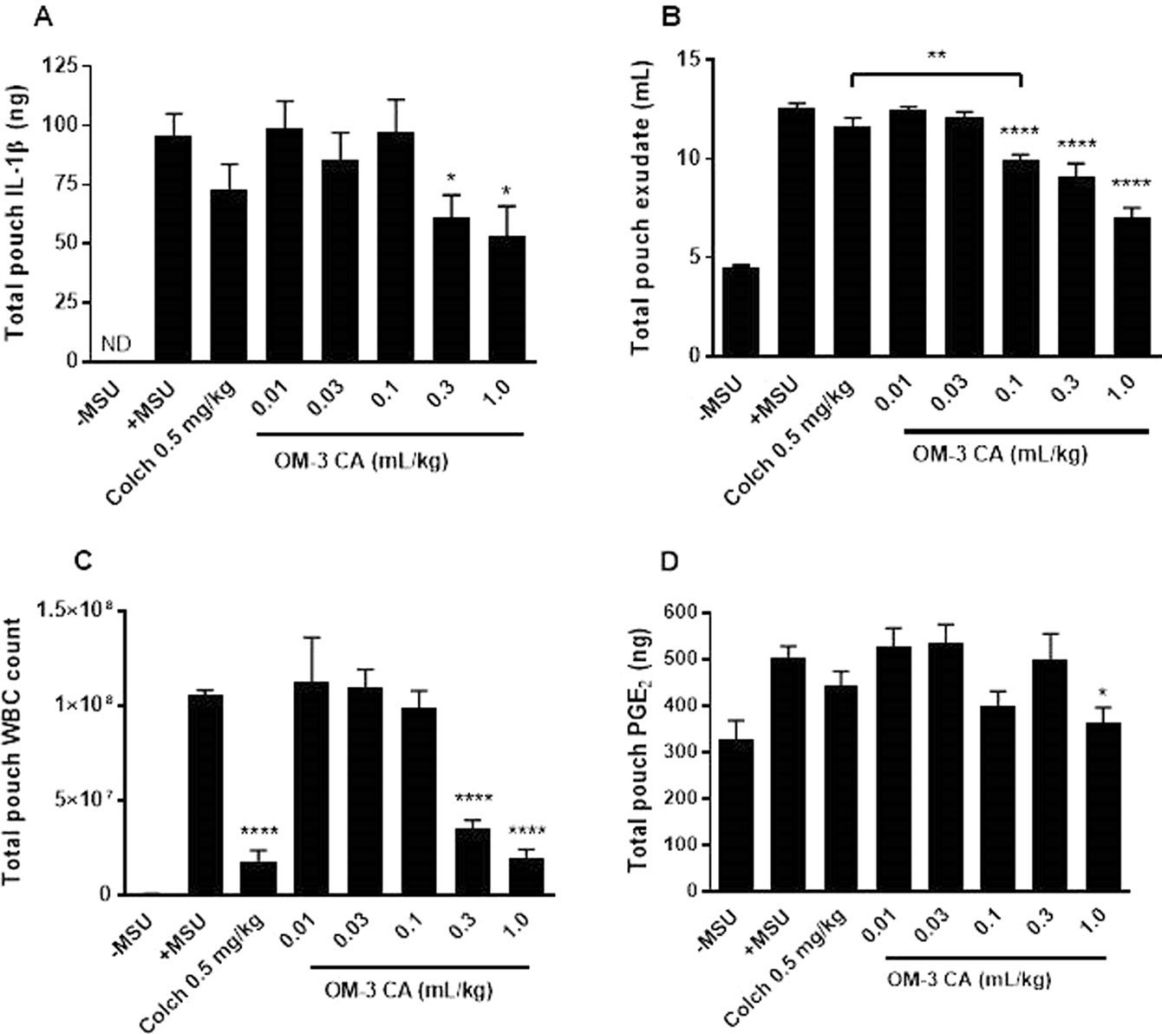
OM-3 CA efficacy in the rat MSU air pouch model tested using four endpoints. (**A**) Mean IL-1β production assayed from the exudate recovered from the air pouches for each treated group (n = 10 per group). (**B**) Mean exudate volume recovered from the air pouches for each treated group. (**C**) Mean number of WBCs recovered from the air pouches for each treated group. (**D**) Pouch exudate PGE_2_ content presented as mean ± standard error of the mean. *P < 0.05 versus + MSU using Dunn’s multiple comparison test (**A** and **D**), **P < 0.01 using Bonferroni’s multiple comparison test versus colchicine (**B**), ***P < 0.001 versus + MSU using Dunnett’s multiple comparison test (**B** and **C**). Colch: colchicine; IL-1β: interleukin-1β; MSU: monosodium urate; ND: not detected; OM-3 CA: omega-3-carboxylic acids; PGE_2_: prostaglandin E_2_; WBC: white blood cell.

Injection of MSU into the rat air pouch caused dramatic increases in WBC infiltration, exudate production, exudate IL-1β content, and exudate PGE_2_ content (Fig. 2). These increases were not inhibited by daily dosing with the 1 mL/kg of the safflower oil vehicle, which contains mainly unsaturated fatty acids but small amounts of OM-3FA, or a PBS control (data not shown). Colchicine is the current first-line treatment for prophylaxis and acute treatment of gout flares^39^, and was used as a positive control. Colchicine strongly reduced WBC infiltration (Fig. 2C) but did not significantly reduce exudate volume or production of IL-1β and PGE_2_. In contrast, OM-3 CA reduced IL-1β and PGE_2_ production, exudate volume, and WBC infiltration in a dose-dependent manner. The overall efficacy of OM-3 CA at amounts of 0.1 mL/kg and higher was superior to that of colchicine in reducing exudate production and equivalent for amounts above 0.1 mL/kg for all other endpoints (Fig. 2). The broad activity of OM-3 CA suggests that multiple anti-inflammatory mechanisms are active *in vivo*.

### OM-3 fatty acids inhibit crystal-mediated WBC infiltration in a dose-dependent manner

**Figure 3:**
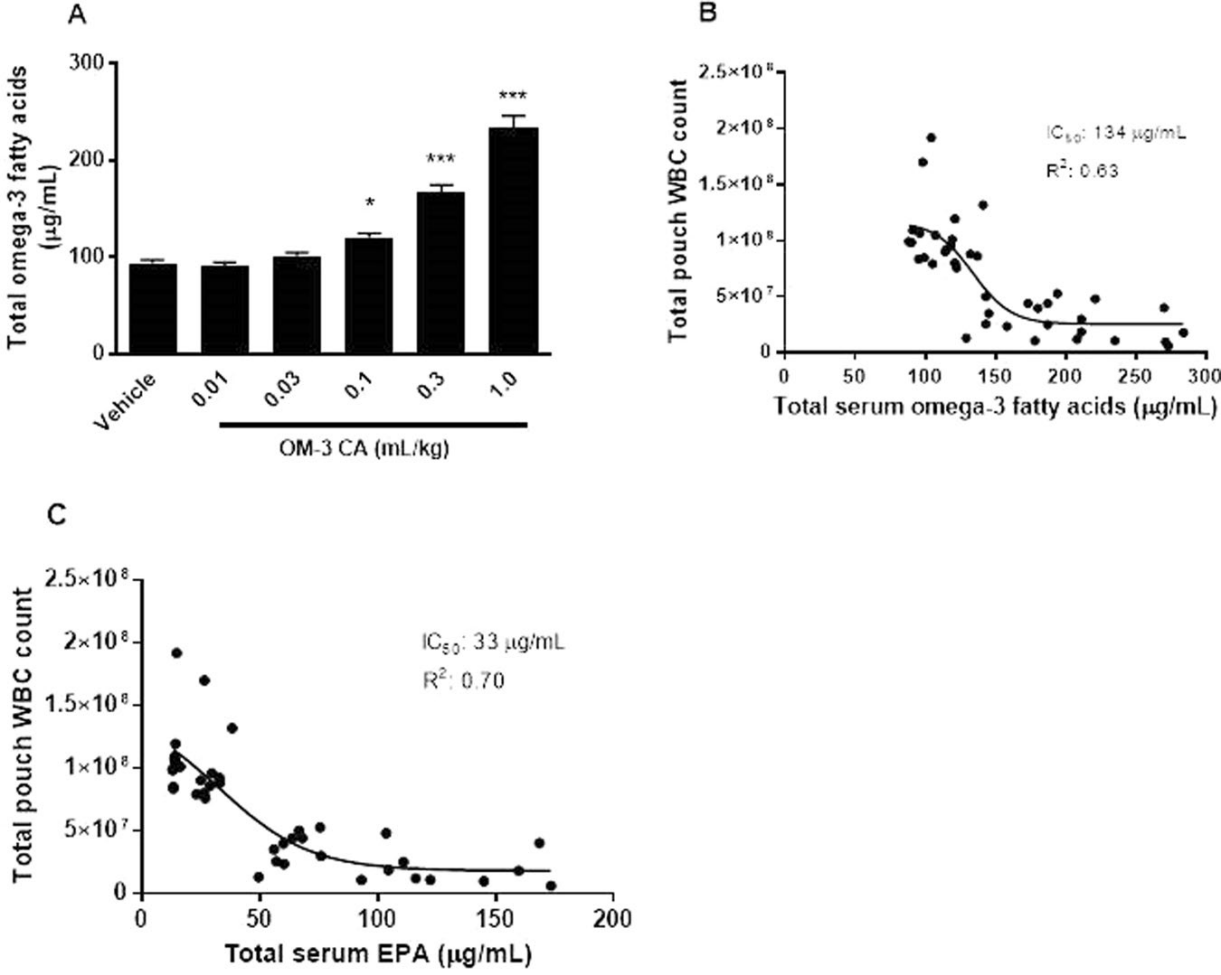
OM-3 CA exposure–response relationships: rat serum omega-3 levels after daily oral dosing with OM-3 CA. (**A**) Total combined docosahexaenoic acid, EPA, docosapentaenoic acid, and α-linolenic acid serum omega-3 levels by OM-3 CA dose. (**B**) OM-3 CA dose groups: 0.03–1.0 mL/kg. Non-linear regression analysis of total pouch WBC count versus serum total OM-3 fatty acids level. (**C**) Non-linear regression analysis of total pouch WBC count versus serum EPA level *P < 0.05, ***P < 0.001 using one-way analysis of variance and Dunnett’s multiple comparison tests versus vehicle. EPA: eicosapentaenoic acid; IC_50_: half-maximal inhibitory concentration; OM-3 CA: omega-3-carboxylic acids; *R*^2^: coefficient of determination; WBC: white blood cell.

In the air pouch study, levels of OM-3 fatty acids in serum were analysed. An exposure–response relationship was observed between total serum OM-3 fatty acid concentration and WBC infiltration in the pouch. OM-3 CA amounts starting from 0.1 mL/kg showed a significant increase over control samples in terms of total OM-3 fatty acid concentration, corresponding to the dose with significant activity in reducing exudate volume, IL-1β concentration, and WBC infiltration (Fig. 3A). Regression analysis of total serum OM-3 fatty acid inhibition data returned an effective IC_50_ of 134 µg/mL for the WBC endpoint, corresponding to an ED_50_ of approximately 0.16 mL/kg OM-3 CA (Fig. 3A and B). Additionally, a regression analysis for individual OM-3 fatty acids showed the best relationship to be between EPA and reduction in WBC infiltration (IC_50_ 33 µg/mL, coefficient of determination [*R*^2^] 0.70) (Fig. 3C). Docosapentaenoic acid (*R*^2^ 0.24) had a weak exposure–response relationship. No relationship was found for DHA and α-linolenic acid, possibly owing to the higher baseline level of these fatty acids in serum (data not shown) and a smaller overall increase in serum levels upon dosing.

### OM-3 CA modifies the progression of inflammation caused by intra-articular injection of MSU

**Figure 4:**
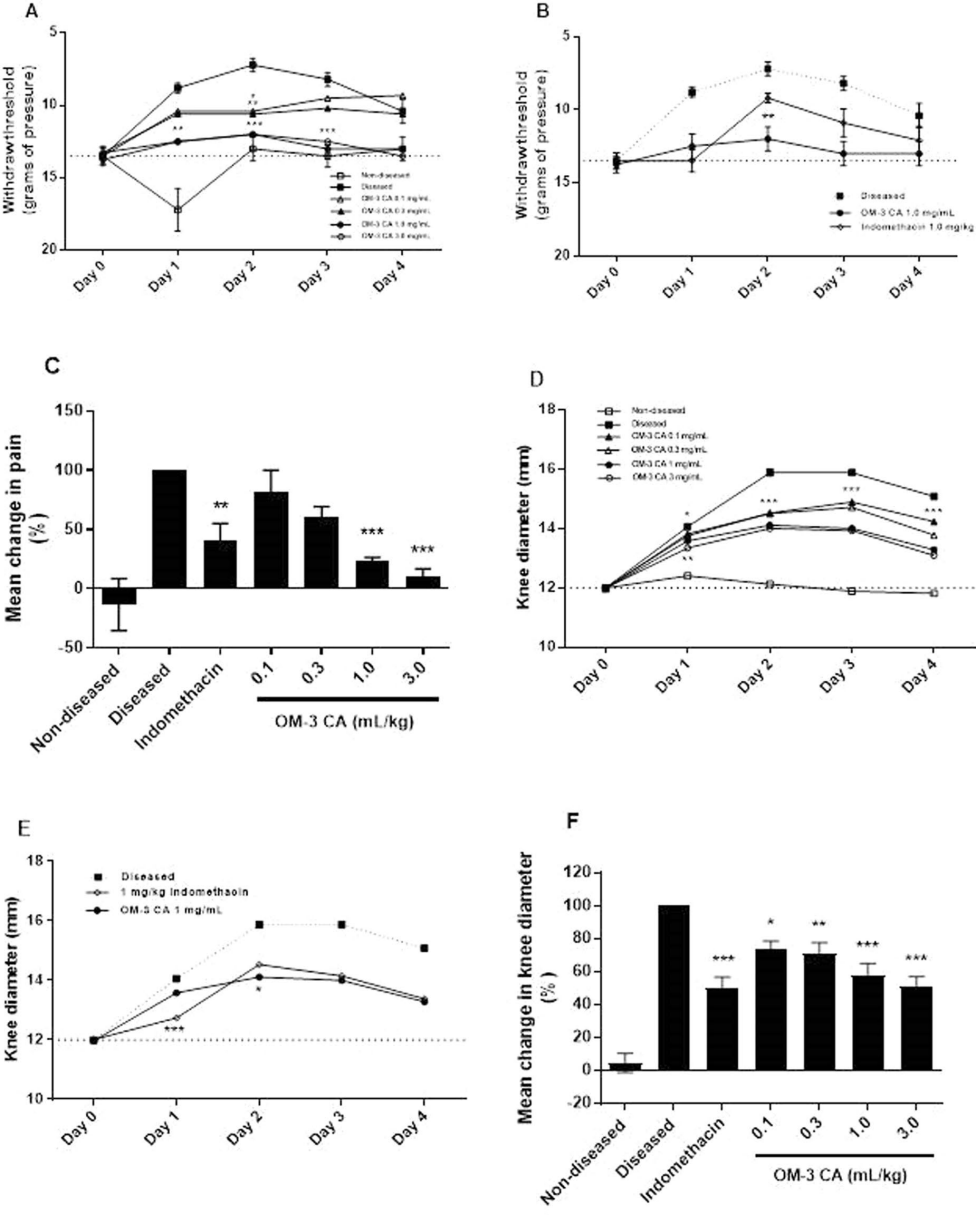
OM-3 CA efficacy in the rat intra-articular knee injection model. (**A**) Daily disease progression as measured by mean withdraw threshold (grams of pressure) over the 4-day study period for OM-3 CA treatment groups. (**B**) Daily disease progression as measured by mean withdraw threshold (grams of pressure) over the 4-day study period for OM-3 CA 1.0 mL/kg versus indomethacin 1.0 mg/kg daily. (**C**) Mean percentage change in pain level over the 4-day study period compared with the diseased group. (**D**) Daily disease progression as measured by knee diameter over the 4-day study period for OM-3 CA treatment groups. (**E**) Daily disease progression as measured by knee diameter over the 4-day study period for OM-3 CA 1.0 mL/kg versus indomethacin 1.0 mg/kg. (**F**) Mean percentage change in knee diameter over the 4-day study period compared with the diseased group. *P < 0.05, **P < 0.01, ***P < 0.001 versus the diseased group using Dunnett’s multiple comparison tests (**A**,**C**,**D**, and **F**) or versus indomethacin using *t*-tests (**B** and **E**). OM-3 CA: omega-3-carboxylic acids.

The rat intra-articular MSU injection model follows the progression of inflammation caused by MSU by measuring pain and swelling over multiple days until self-resolution. This is important because some anti-inflammatory therapeutics only delay disease progression or inhibit resolution of inflammation^40^. Pain was quantified by assessing the mechanical allodynia threshold. In this model, the NSAID indomethacin was given orally every day as a positive control. This control is preferred over colchicine because of the toxicity associated with multiple high amounts of colchicine^41–43^. In the vehicle-treated group, the intra-articular MSU injection caused increases in pain and swelling within 1 day that peaked at day 2, after which inflammation began to resolve (Fig. 4A and D). OM-3 CA inhibited pain and swelling in a dose-dependent manner. The onset of effect was seen at the first time point evaluated (day 1) and was sustained throughout the 4-day observational period (Fig. 4A and D). The mean reduction in pain over the 4-day period was significant at the two highest amounts of OM-3 CA (Fig. 4C). The mean reduction in knee mean diameter was statistically significant at all amounts and was amount ordered (Fig. 4F). In the indomethacin-treated group, there was potent suppression of pain and swelling at day 1, but an increase in both endpoints from day 1 to day 2. This resulted in a superior day 2 effect of OM-3 CA on both withdrawal threshold and knee diameter compared with indomethacin (Fig. 4B and E). The two high amounts of OM-3 CA (1 mL/kg and 3 mL/kg) and indomethacin resulted in comparable reductions in mean knee diameter (Fig. 4F). A probable reason for the loss of efficacy of indomethacin is that other pro-inflammatory mechanisms induced by MSU (e.g. release of mature IL-1β) are able to overcome the specific inhibition of prostaglandin synthesis, which results in swelling and pain. Additionally, upregulation of COX-2 expression by MSU may overcome the ability of indomethacin to fully inhibit prostaglandin synthesis in monocytes^38^.

### OM-3 CA is more active than over-the-counter OM-3-triglycerides

**Figure 5:**
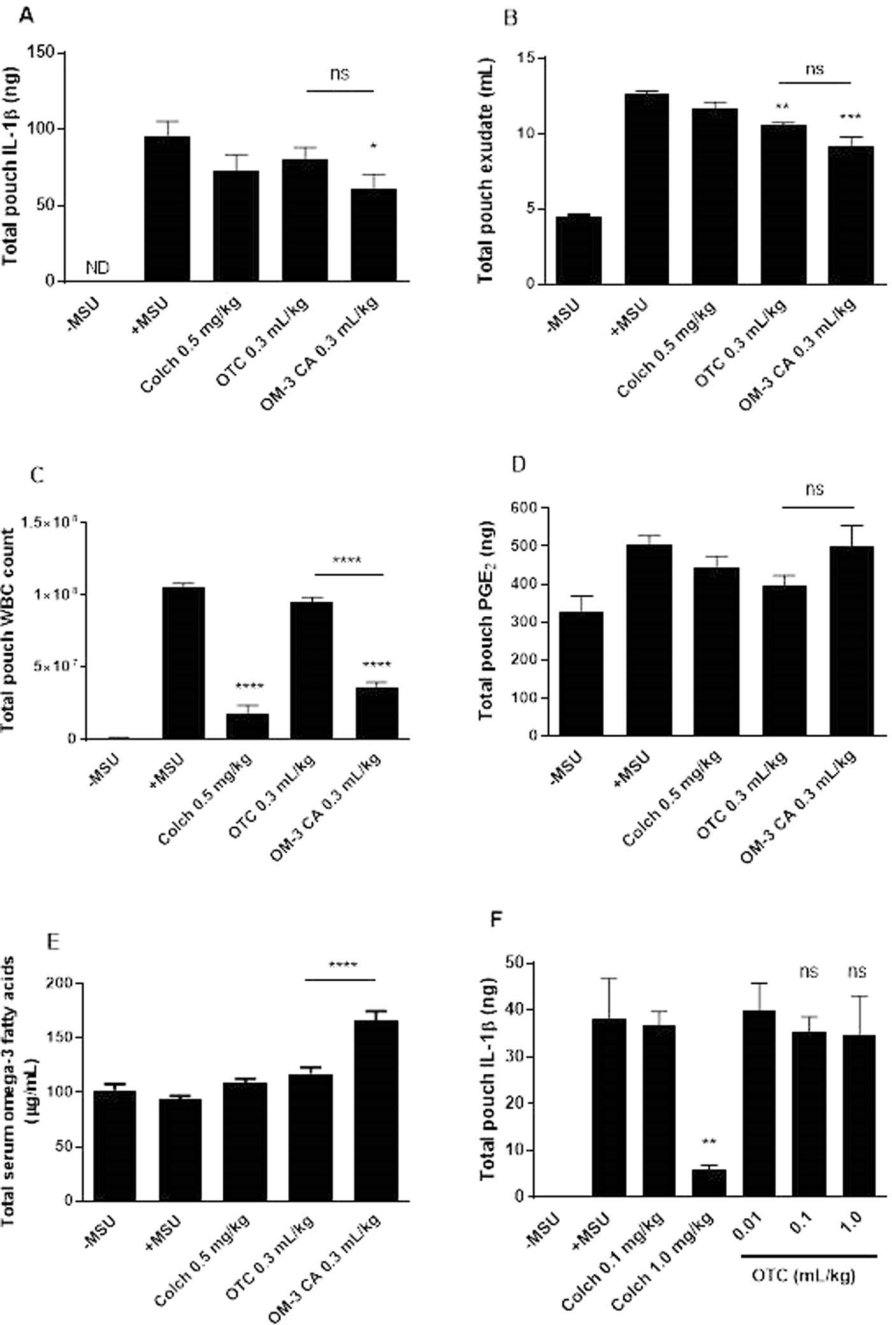
Comparison of OM-3 CA with OTC OM-3-triglycerides, both at amounts of 0.3 mL/kg/day, in the rat MSU air pouch model. (**A**) Mean IL-1β production assayed from the exudate recovered from the air pouches for each treated group (n = 10 per group). (**B**) Mean exudate volume recovered from the air pouches for each treated group. (**C**) Mean number of WBCs recovered from the air pouches for each treated group. (**D**) Pouch exudate PGE_2_ content presented as mean ± standard error of the mean. ns, not significant. *P < 0.05 versus + MSU using Kruskal–Wallis test with Dunn’s multiple comparison test (**A** and **D**), **P < 0.01, ***P < 0.001, ****P < 0.0001 versus + MSU by one-way ANOVA with Bonferroni’s multiple comparison test. (**E**) Total serum omega-3 of comparator groups. ****P < 0.0001 using one-way ANOVA with Bonferroni’s multiple comparison tests. (**F**) IL-1β endpoint on titrations of OTC OM-3-triglycerides up to 1.0 mL/kg. ns, not significant versus + MSU. **P < 0.01 versus + MSU using Kruskal–Wallis tests with Dunn’s multiple comparison tests. ANOVA: analysis of variance; Colch: colchicine; IL-1β: interleukin-1β; MSU: monosodium urate; ND: not detected; OM-3: omega-3; OM-3 CA: omega-3-carboxylic acids. OTC: over-the-counter OM-3 triglycerides; PGE_2_: prostaglandin E_2_.

To test the hypothesis that purity, quantity, and bioavailability are important factors in the anti-inflammatory effects of OM-3 fatty acid treatment, OM-3 CA was compared with over-the-counter (OTC) OM-3-triglycerides in the MSU air pouch model. Equal volumes of each drug were given at approximately 2 × ED_50_ of OM-3 CA for the WBC endpoint (0.3 mL/kg) for 7 days prior to MSU challenge. OM-3 CA significantly decreased IL-1β, whereas OM3-OTC did not (Fig. 5A); however both formulations decreased pouch exudate volume (Fig. 5B). OM3-CA significantly decreased WBC infiltration, while OM3-OTC had no effect as compared to the MSU control group (Fig. 5C). Neither drug significantly inhibited PGE_2_ production (Fig. 5D). Serum analysis of total OM-3 fatty acid exposure revealed approximately threefold higher exposure in the OM-3 CA group than in the OTC group (Fig. 5E). Increasing amounts of OTC OM-3-triglycerides increased the exposure and improved all endpoints (data not shown) with the exception of pouch IL-1β, where no activity was seen at any amount tested (Fig. 5F). Despite this increase in efficacy for most endpoints, no exposure–response relationship between OTC OM-3-triglycerides and the endpoints was observed, possibly owing to the limited amounts tested.

### Palmitic acid can play a pro-inflammatory role

**Figure 6:**
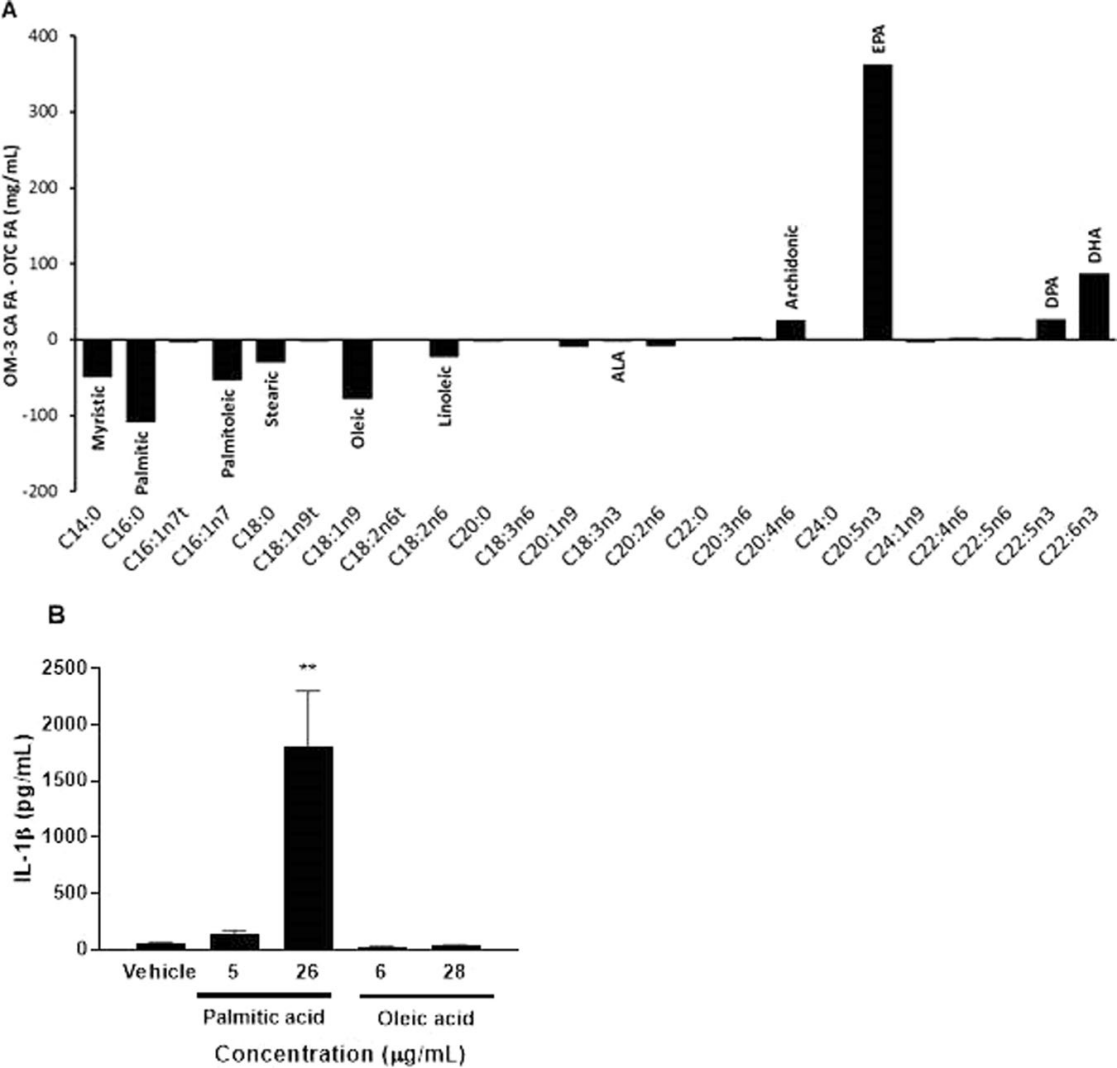
Dosing solution fatty acid profiles and macrophage activation by fatty acids. (**A**) Dosing solution fatty acid profiles as determined by gas chromatography. The difference between OM-3 CA and OTC dosing solutions is shown as a mg/mL difference in individual fatty acid content. (**B**) Macrophage activation by fatty acids. THP-1 macrophages were incubated with either 20 µM (5 or 6 µg/mL) or 100 µM (26 or 28 µg/mL) fatty acids for 26 hours, and IL-1β production was measured. The mean and standard error of the mean are shown from two independent experiments. **P < 0.01 versus vehicle using Student’s t-test. ALA: α-linolenic acid; DHA: docosahexaenoic acid; DPA: docosapentaenoic acid; EPA: eicosapentaenoic acid; FA: fatty acids; IL-1β: interleukin-1β; OM-3 CA: omega-3-carboxylic acids; OTC: over-the-counter.

To understand the differential effects on the IL-1β endpoint observed between OM-3 CA and OTC OM-3-triglycerides, we analysed the fatty acid profiles of the dosing solutions and tested the effects of individual fatty acids on macrophage activation. The OM-3 CA formulation contained higher amounts of total OM-3 fatty acids than the OTC solution, which explains the higher serum levels of these fatty acids (Table 1 and Fig. 6A). The OTC solution had higher concentrations of some saturated fatty acids, mainly myristic (C14:0), palmitic (C16:0), and stearic (C18:0) acids (Table 1). We evaluated the ability of palmitic (C16:0) and oleic (C18:1n9) acids to activate macrophages because these fatty acids had the largest individual differences with the OM-3 CA dosing solutions. THP-1 macrophages were incubated for 24 hours with fatty acids (20 µM or 100 µM) or vehicle; supernatants were then removed and assayed for mature IL-1β. Incubation with palmitic acid, but not oleic acid, was associated with a dose-dependent increase in IL-1β production (Fig. 6B). These results suggest that palmitic acid alone may diminish potential beneficial effects of OM-3 fatty acids on IL-1β production, and that drug purity of fatty acid mixtures is important to achieve an anti-inflammatory benefit.

**Table 1:** Fatty acid profile of vehicle and dosing solutions. OM-3 CA: omega-3-carboxylic acids; OTC, over-the-counter.

Class	Common name	Lipid name	Vehicle (mg/mL)	OTC (mg/mL)	OM-3 CA (mg/mL)
Omega-3	α-linolenic	C18:3n3	1.4	5.5	3.0
	Eicosapentaenoic	C20:5n3	0.0	129.8	491.7
	Docosapentaenoic n-3	C22:5n3	0.0	15.7	42.4
	Docosahexaenoic	C22:6n3	0.1	85.1	171.9
Omega-6	Linoelaidic	C18:2n6t	0.4	2.3	1.0
	Linoleic	C18:2n6	134.7	27.5	4.9
	γ-linolenic	C18:3n6	0.0	2.3	1.8
	Eicosadienoic	C20:2n6	0.4	22.6	14.7
	Dihomo-γ-linolenic	C20:3n6	0.0	1.3	4.2
	Arachidonic	C20:4n6	0.0	6.9	32.1
	Docosatetraenoic	C22:4n6	0.0	0.6	2.2
	Docosapentaenoic n-6	C22:5n6	0.0	3.1	5.4
Omega-7	Palmitelaidic	C16:1n7t	0.1	3.9	0.7
	Palmitoleic	C16:1n7	0.6	57.3	3.7
Omega-9	Elaidic	C18:1n9t	0.4	2.2	0.3
	Oleic	C18:1n9	651.5	88.1	9.9
	Eicosenoic	C20:1n9	2.5	9.2	0.3
	Nervonic	C24:1n9	1.5	3.6	0.5
Saturated fatty acids	Myristic	C14:0	0.7	50.9	1.6
	Palmitic	C16:0	42.8	109.6	0.8
	Stearic	C18:0	16.5	30.7	1.1
	Arachidic	C20:0	3.4	4.1	2.3
	Behenic	C22:0	2.8	0.5	0.6
	Lignoceric	C24:0	1.6	0.2	0.1

## Discussion

Current anti-inflammatory therapeutic interventions in crystal-mediated diseases inhibit pro-inflammatory prostanoid and cytokine production and reduce cell mobility to achieve reductions in pain and swelling, and ultimately flare resolution^39,44,45^. Crystal-associated inflammation is mediated by activation of the NLRP3 inflammasome in tissue-resident phagocytes. This triggers an auto-inflammatory cascade, which involves many aspects of the innate immune system. OM-3 fatty acids have been shown to modulate IL-1β induction and reduce the synthesis of pro-inflammatory cytokines and PGs both *in vitro* and *in vivo*, making them an attractive option for treatment of inflammatory diseases. Despite the mechanistic rationale, there is a lack of preclinical and clinical studies of OM-3 fatty acids in crystal-mediated diseases such as gout. This may be due to the relatively small effects observed in interventional studies conducted in patients with RA, generating concern that the efficacy of OM-3 fatty acids will not be sufficient to block acute gout flares or to modify progression of an active flare^15^. The mechanism of inflammation in RA is distinct from that in gout, involving the adaptive immune system and different cytokine signalling pathways. As such, potent blockade of IL-1β signalling by biologics has shown only a modest clinical benefit in RA^46,47^. In contrast, gout and other crystal-mediated diseases depend heavily on the activation of the innate immune system, including transduction of IL-1β signalling and PG synthesis. Thus, treatment with OM-3 fatty acids should engage the key pathways active in the disease process, with the potential for enhanced efficacy.

An earlier study demonstrated that dietary sources of EPA were able to block the fluid phase and PGE_2_ induction of crystal inflammation but did not affect the WBC recruitment phase^23^. We showed that therapeutic amounts of OM-3 CA can potently reduce levels of multiple MSU-induced inflammatory markers, including exudate production, WBC recruitment, and induction of IL-1β and PGE_2_. Additionally, OM-3 CA dose-dependently reduced knee pain and swelling caused by intra-articular injection of MSU. OM-3 CA treatment both blocked acute inflammation and modified the course of the disease over an extended duration in a manner distinct from that of NSAIDs.

The two animal models in this study used the relevant first-line therapies as positive controls, which have different anti-inflammatory modes of action. Colchicine preferentially inhibited WBC infiltration but had little effect on exudate or pro-inflammatory mediator production. Indomethacin blocked initial pain and swelling, but on continued dosing there were significant increases in each endpoint. Possible loss of activity may be due to reduced inhibition of IL-1β signaling or MSU-mediated upregulation of COX-2 signaling^35^, which suggests a lack of disease resolution. By contrast, OM-3 CA produced dose-dependent inhibition across multiple inflammatory markers, possibly owing to effective engagement with multiple key inflammation pathways.

Our findings support the theory of an anti-inflammatory threshold for OM-3 fatty acid-based therapies, highlighting the importance of having sufficiently high quantity and purity of the drug substance in order to achieve full efficacy. The fatty acid compositions of OM-3 CA and the OTC OM-3-triglyceride dosing solutions were different. The OTC compound did not inhibit IL-1β production, even under conditions in which total OM-3 fatty acid exposure was equal to that achieved with OM3-CA. However, there was no difference between the effects of OM3-CA and OM3-OTC on IL-1β levels, indicating that mediators other than IL-1β may play a role in the marked difference in effect between the formulations on WBC infiltration. Impurities present in the OTC preparation, specifically palmitic acid, can induce IL-1β and IL-6 production and may explain the lack of effect on this endpoint *in vivo*^48–50^. This finding is consistent with the idea that dietary saturated fatty acids and OM-3 fatty acids have opposing roles in regulating NLRP3 activity in hepatocytes^51^, and may also explain the lack of effect of the OTC OM-3-triglyceride formulation on IL-1β suppression in the MSU air pouch model. Interestingly, palmitic acid-induced IL-6 and IL-8 production in gingival fibroblasts was suppressed by DHA but not EPA, indicating that the two major OM-3 fatty acids may have different anti-inflammatory effects^50^. Further work is needed to clarify the respective roles of DHA and EPA on crystal-induced inflammation.

Study limitations include the lack of testing of highly purified OM-3-triglyceride and OM-3 ethyl ester formulations for anti-IL-1β activity *in vivo*. These data would shed light on the question of impurities versus differences in dosed OM-3 fatty acid species as the mediator of anti-IL-1β activity. Longer and shorter dosing intervals might also allow the differential effects between OM-3 CA and other formulations of OM-3 fatty acids to be explored further.

Despite our study limitations, the data indicate that OM-3 CA has anti-inflammatory effects with a strong exposure–response relationship that could be beneficial in the prevention and treatment of crystal arthritis, with potential applications in other IL-1β-mediated diseases that engage the innate immune system.
